# Univariable and Multivariable Two-Sample Mendelian Randomization Investigating the Effects of Leisure Sedentary Behaviors on the Risk of Lung Cancer

**DOI:** 10.3389/fgene.2021.742718

**Published:** 2021-11-24

**Authors:** Haoxin Peng, Xiangrong Wu, Yaokai Wen, Yiyuan Ao, Yutian Li, Wenhui Guan, Jinsheng Lin, Caichen Li, Hengrui Liang, Jianxing He, Wenhua Liang

**Affiliations:** ^1^ Department of Thoracic Oncology and Surgery, China State Key Laboratory of Respiratory Disease and National Clinical Research Center for Respiratory Disease, the First Affiliated Hospital of Guangzhou Medical University, Guangzhou, China; ^2^ Nanshan School, Guangzhou Medical University, Guangzhou, China; ^3^ School of Medicine, Tongji University, Shanghai, China; ^4^ Department of Medical Oncology, Shanghai Pulmonary Hospital, Tongji University Medical School Cancer Institute, Tongji University School of Medicine, Shanghai, China; ^5^ Medical Oncology, the First People’s Hospital of Zhaoqing, Zhaoqing, China

**Keywords:** leisure sedentary behaviors, lung cancer, Mendelian randomization, cancer prevention, single-neucleotide polymorphism

## Abstract

Leisure sedentary behaviors (LSB) are widespread, and observational studies have provided emerging evidence that LSB play a role in the development of lung cancer (LC). However, the causal inference between LSB and LC remains unknown.

**Methods:** We utilized univariable (UVMR) and multivariable two-sample Mendelian randomization (MVMR) analysis to disentangle the effects of LSB on the risk of LC. MR analysis was conducted with genetic variants from genome-wide association studies of LSB (408,815 persons from UK Biobank), containing 152 single-nucleotide polymorphisms (SNPs) for television (TV) watching, 37 SNPs for computer use, and four SNPs for driving, and LC from the International Lung Cancer Consortium (11,348 cases and 15,861 controls). Multiple sensitivity analyses were further performed to verify the causality.

**Results:** UVMR demonstrated that genetically predisposed 1.5-h increase in LSB spent on watching TV increased the odds of LC by 90% [odds ratio (OR), 1.90; 95% confidence interval (CI), 1.44–2.50; *p* < 0.001]. Similar trends were observed for squamous cell lung cancer (OR, 1.97; 95%CI, 1.31–2.94; *p* = 0.0010) and lung adenocarcinoma (OR, 1.64; 95%CI 1.12–2.39; *p* = 0.0110). The causal effects remained significant after adjusting for education (OR, 1.97; 95%CI, 1.44–2.68; *p* < 0.001) and body mass index (OR, 1.86; 95%CI, 1.36–2.54; *p* < 0.001) through MVMR approach. No association was found between prolonged LSB spent on computer use and driving and LC risk. Genetically predisposed prolonged LSB was additionally correlated with smoking (OR, 1.557; 95%CI, 1.287–1.884; *p* < 0.001) and alcohol consumption (OR, 1.010; 95%CI, 1.004–1.016; *p* = 0.0016). Consistency of results across complementary sensitivity MR methods further strengthened the causality.

**Conclusion:** Robust evidence was demonstrated for an independent, causal effect of LSB spent on watching TV in increasing the risk of LC. Further work is necessary to investigate the potential mechanisms.

## Introduction

Lung cancer (LC) is a leading health problem worldwide and accounts for nearly a quarter of cancer deaths in the United States. Regarding sex, LC ranks as the second most common cancer for both males and females (Siegel et al., 2021). To decrease the disease burden of LC, examining the potential risk factors is of vital importance. Taking cigarette smoking, a well-established risk factor for LC, as an example, the publication of tobacco control policies greatly decreased the cigarette smoking rates, and the morbidity and mortality of LC have declined subsequently (Carter et al., 2015). In addition to cigarette smoking, epidemiologists have identified numerous risk factors for LC in the recent decades, of which lifestyle behavior is one of the research focuses—for instance, excessive alcohol consumption has been reported to increase the odds of LC by 94% in a recent study (Larsson et al., 2020). On the contrary, dietary fiber, yogurt consumption, and physical activity (PA) are correlated with a lower risk of LC (Schmid et al., 2016; Yang et al., 2020).

Sedentary behavior (SB) is widespread in all walks of life, including recreation like watching television (TV) and computer, occupations like driving and sitting at a counter, and social activities like a meeting. SB is a broad categorical name describing various types of human behaviors featuring an energy expenditure ≤1.5 metabolic equivalents (METs) while reclining, sitting, or in a lying posture (van de Vegte et al., 2020). Notably, SB includes behaviors for leisure, that is, leisure sedentary behaviors (LSB), like watching TV, for occupation, and so on (Katzmarzyk et al., 2019). SB gradually comes to be considered a major health problem due to its high prevalence, and more importantly, it is correlated with increased risks of cancer and all-cause mortality (Basterra-Gortari et al., 2014). Possible mechanisms underlying the attribution of SB to cancers, like increased insulin resistance and chronic inflammation marked by higher concentrations of C-reactive protein (CRP), have been proposed in recent years (Kerr et al., 2017). A large meta-analysis consisting of 43 observational studies found that prolonged SB spent on TV watching, which is often used as a proxy of SB since it is easy to recall, was positively associated with LC, colorectal cancer, and endometrial cancer (Schmid and Leitzmann, 2014). Nevertheless, the potential effects of PA and SB might tangle in these observational studies—for instance, the time spent on SB would be attenuated in those who are physically active compared with those who are physically inactive (Jiang et al., 2019). Consequently, previous studies were unlikely to fully disentangle the independent effects of SB on the risk of LC. Moreover, previous studies obtained data on the measurement of SB through questionnaires and self-reports, making recall bias unavoidable. Therefore, whether the effect of SB on the risk of LC is independent and whether the causal inference from SB to LC is true remains unclear.

Mendelian randomization (MR) is a novel methodological advance for investigating the causal inference between an exposure and an outcome utilizing summary genetic data from genome-wide association studies (GWASs) (Sekula et al., 2016). The MR approach is based on Mendel’s second law which states that genetic variants, like single-nucleotide polymorphism (SNP), are randomly distributed at conception, which are generally independent of environmental risk factors and satisfy the chronological order for probing causal inference (Smith and Ebrahim, 2004). MR was conducted based on three assumptions (Siegel et al., 2021): the instrumental variable (IV) is robustly associated with exposure (Carter et al., 2015), the IV is independent of any confounders, and (Larsson et al., 2020) the IV does not affect the outcome other than through the exposure (Emdin et al., 2017). Consequently, the causal effect from an exposure to an outcome can be estimated by employing MR analysis, which avoids potential limitations, like confounding bias, measurement error, and reverse causation, that are common in conventional observational studies since the nature of SB traits is complex and various factors are likely to affect SB, like body mass index (BMI) and educational attainment (van de Vegte et al., 2020). The SNP, utilized as a proxy of SB, may directly affect the LC risk *via* educational attainment/BMI rather than being mediated *via* SB. To avoid such pleiotropic effects which may violate the third MR assumption, the multivariable MR (MVMR) approach was introduced (Sanderson et al., 2019). The MVMR method simultaneously evaluates the effects of two or more risk factors that share a set of overlapping SNPs to ensure that the direct effect of each exposure on an outcome is not mediated by other factors (Burgess and Thompson, 2015). MVMR analysis has been carried out in many studies. Recently, researchers found that per 1.5-h increase in LSB of watching TV raised the risk of coronary artery disease by 44%, which was partially independent of educational attainment and BMI through the MVMR approach (van de Vegte et al., 2020). However, there are no related studies focusing on the relationship between LSB and LC risk.

In the present study, we pay particular attention to disentangle the independent effects of LSB on the risk of LC, overall and by histotype, including squamous cell lung cancer (LUSC) and lung adenocarcinoma (LUAD), using univariable MR (UVMR) and multivariable MR (MVMR) methods.

## Materials and Methods

### GWAS Summary Data

Genetic summary data from large consortia for LCB as well as International Lung Cancer Consortium (ILCCO) were publicly available on the MR-Base platform, an analytical platform and database for MR (http://www.mrbase.org/) (Hemani et al., 2018).

### Genetic Instruments for SB

**FIGURE 1:**
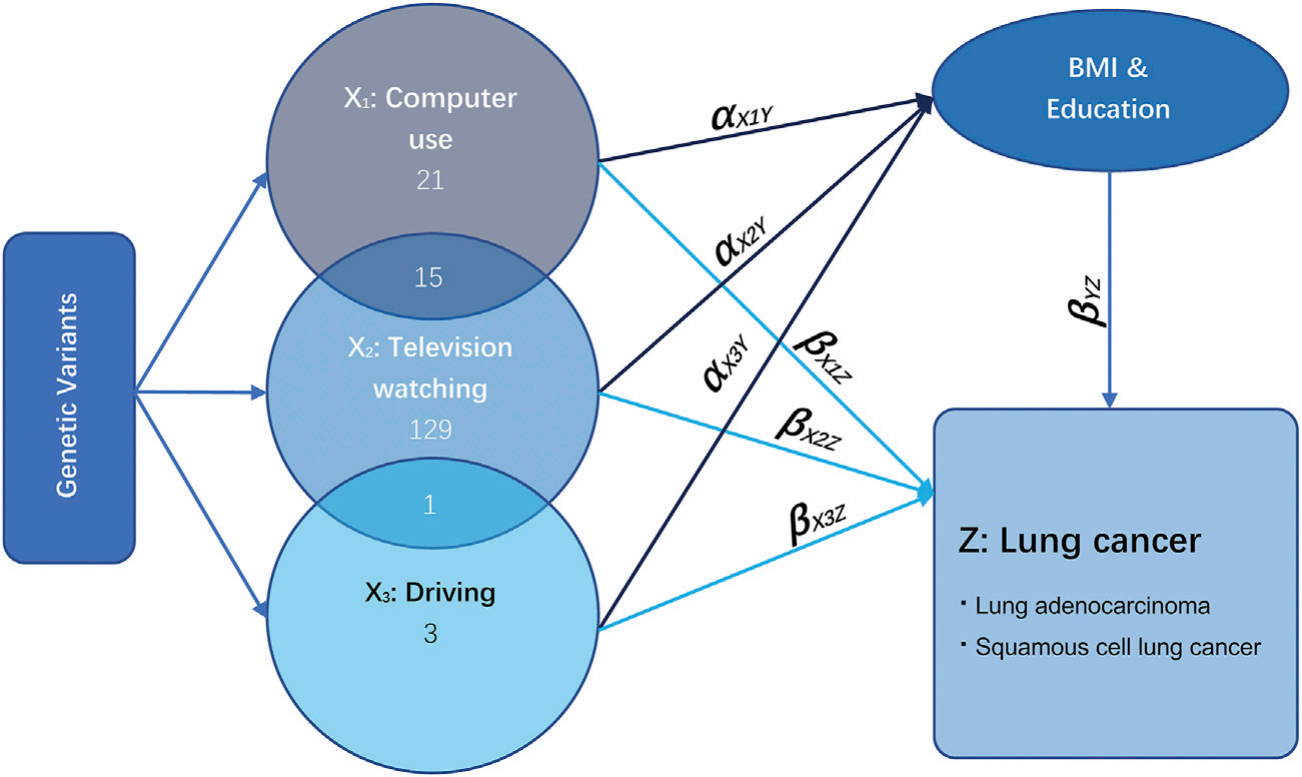
Illustrative diagram of multivariable Mendelian randomization assumptions. Taking the exposure television watching (X_2_) as an example, the direct effect of television watching on lung cancer is the effect television watching has on lung cancer not *via* any other exposure variables, which is equal to β_X2Z_. The total effect of television watching on lung cancer is the effect of television watching on lung cancer directly plus the effect of television watching on lung cancer *via* education and BMI, which is equal to β_X2Z_ + α_X2Y_β_YZ_.

We identified genetic instruments for LSB using outcomes from the largest and latest available GWASs meta-analysis, including TV watching, computer use, and driving behaviors, which combined the summary data from the UK Biobank (van de Vegte et al., 2020). The study included 422,218 individuals of European ancestry The average age was 57.4 years old [standard deviation (SD), 8.0 years old] during first assessment, and male consisted 45.7% of the participants (van de Vegte et al., 2020). In brief, the mean reported time for daily leisure TV watching was 2.8 h (SD, 1.5 h), 1.0 h (SD, 1.2 h) for leisure computer use, and 0.9 h (SD 1.0 h) for driving. Eventually, 193 variants in 169 loci correlated with one or more sedentary traits were revealed in the study, the large majority of which, including 152 independent variants in 145 loci, were correlated with leisure TV watching, 37 independent variants in 36 loci were for leisure computer use, and four independent variants in four loci were for leisure driving. Notably, 15 overlapping loci were shared between TV watching and computer use. TV watching also had one shared locus with driving, while driving showed no overlap with computer use (Figure 1). We also manually searched whether LSB-related SNPs have secondary phenotypes other than LSB in the NHGRI-EBI catalog of human genome-wide association studies (https://www.ebi.ac.uk/gwas/), the PhenoScanner (http://www.phenoscanner.medschl.cam.ac.uk/), and the National Center for Biotechnology Information (https://www.ncbi.nlm.nih.gov/) databases (Swerdlow et al., 2016; Phelan et al., 2017).

The IVs for MR analyses were selected based on the following criteria: (i) *p*-value less than the genome-wide significance threshold of 5 × 10^−8^, (ii) secondly conjugate linkage disequilibrium analysis not exceeding the limited *R* ^2^ value of 0.001, and (iii) not being palindromic with intermediate allele frequencies.

### Genetic Associations of SNPs With LC Risk

Summary statistics on LC risk, including effect estimates (beta) and standard errors for instrumental SNPs, were retrieved based on 11,348 LC cases and 15,861 controls from the ILCCO (Table 1) (Wang et al., 2014), which owns considerable genetic data from ongoing LC case–control and cohort studies. A subgroup of LC cases was constructed based on the histotypes of non-small cell lung cancer (NSCLC), including LUAD and LUSC, to provide more concrete results for the corresponding populations. The analysis was restricted to participants from European ancestries, in correspondence with IVs for SB (Wang et al., 2014; van de Vegte et al., 2020), to minimize bias at the chromosome level.

**TABLE 1:** Details of epidemiological individual-level data of lung cancer from the International Lung and Cancer Consortium (ILCCO) included in Mendelian randomization analyses.

Trait	First author	Consortium	Number of cases	Number of controls	Proportion of cases	Population
Lung cancer	Y Wang	ILCCO	11,348	15,861	0.42	European
Lung adenocarcinoma	Y Wang	ILCCO	3,422	14,894	0.23	European
Squamous cell lung cancer	Y Wang	ILCCO	3,275	15,038	0.22	European

### MVMR Analysis

The relationship between LSB (including TV watching, computer use, and driving) and LC is illustrated in Figure 1. According to the GWAS of LSB assessment and description, high genetic correlations were observed between SB and other traits, including BMI and education (van de Vegte et al., 2020). Thus, the remarkable pleiotropic effect of education and BMI required close attention. X_1_ represents the exposure phenotype of computer use, X_2_ represents TV watching, and X_3_ stands for driving. Several loci were observed to be shared between them. The direct effect of LSB on LC is the effect that is independent from BMI and education attainment, which is equal to β_X1Z_, β_X2Z_, and β_X3Z_. The total effect is the effect of LSB on LC directly plus the effect of leisure sedentary on LC *via* BMI and education, equaling to β_(X1Z+X2Z+X3Z)_ + α_(X1Y+X2Y+X3Y)*_β_YZ_ (Burgess and Thompson, 2015). UVMR analysis for LSB with shared loci with BMI and education was performed to estimate the total causal effects using a random-effects inverse variance-weighted (IVW) Wald-type estimator for deriving a MR estimate of multiple IVs (Lawlor et al., 2008).

To investigate the direct effects of LSB on LC, we performed MVMR analysis, an extension of UVMR which enables to detect causal effects of multiple risk factors jointly (Sanderson et al., 2019). MVMR considers that the SNPs used in the MR analyses are also associated with education attainment and BMI (van de Vegte et al., 2020). The SNPs used to conduct MVMR were combinations of IVs of each exposure. The exclusion criteria of SNPs were consistent with UVMR analyses. Summary MR estimates of the LSB-LC were conducted using the “TwoSampleMR” package (version 0.5.4) (Hemani et al., 2018) in R (version 3.6.2).

### Sensitivity Analysis

A two-sample conditional F-statistic can test whether the genetic variants strongly predict each exposure conditional on the other exposures included in a MVMR model, while the general F-statistic is incapable (Sanderson et al., 2021). In order to evaluate the strength of SNP exposure conditional on other exposures, the two-sample conditional F-statistics were estimated using the online platform provided by Brion *et al*. (http://glimmer.rstudio.com/kn3in/mRnd/) (Brion et al., 2013; Sanderson et al., 2019). By omitting one SNP sequentially and examining variation in causality and overall IVW estimation, leave-one-out analysis was employed for evaluating whether MR estimation was driven or biased by a single SNP. Weighted median analysis was performed, allowing up to 50% of information from variants to violate MR assumptions (Sekula et al., 2016). As a pleiotropy test, MR-Egger regression was also carried out by accessing the effects of global pleiotropy (Bowden et al., 2015). Furthermore, MR pleiotropy residual sum and outlier (MR-PRESSO) test was used to detect pleiotropy as well, during which outliers can be identified and pleiotropic effects can be detected. The association without the outliers would be re-analyzed to correct possible pleiotropic effects (Verbanck et al., 2018). To evaluate whether any single IV was biasing the results and to check for the consistency of MR assumptions, the heterogeneity estimated by Cochran’s Q test was utilized (Bowden et al., 2017; Emdin et al., 2017). The *I* ^2^ index in the inverse variance-weighted method was also calculated to estimate the heterogeneity, which overcomes the problem of Cochran’s Q test that it may suffer from low statistical power when the number of estimates to be pooled is small (Greco M et al., 2015). The *I* ^2^ index equals to ( $\frac{Q-df}{Q}$ ) * 100%, and the heterogeneity was regarded as significant if *I* ^2^ >25%. Regarding the formula, “*Q*” is the quantitative value of Cochran’s Q test, and “*df*” is the degree of freedom which equals to the number of instrumental variables used minus one.

Besides this, additional MR analyses were also conducted to further investigate the mediating effects from a genetically predisposed longer time spent on LSB to LC, including smoking (ever *vs*. never smoked; former *vs*. current smoker; cigarettes smoked per day) and alcohol consumption (previous *vs*. never) (Table 2) (Censin et al., 2019; Larsson et al., 2020). Both the exposure-mediator effects and the mediator-outcome effects were estimated in the separate analyses.

**TABLE 2:** Details of studies included in the Mendelian randomization analysis for potential mediators between leisure sedentary behaviors and lung cancer.

Traits	First author	Consortium	Study participants	Year	PubMed ID
Former *vs*. current smoker	Furberg	TAG	41,969	2010	20418890
Ever *vs*. never smoked	Furberg	TAG	74,035	2010	20418890
Cigarettes smoked per day	Furberg	TAG	68,028	2010	20418890
Alcohol drinker status: previous	Neale	Neale Lab	336,965	2017	10894596
Alcohol drinker status: never	Neale	Neale Lab	336,965	2017	10894596

TAG, Tobacco and Genetics consortium.

## Results

### Genetic Instruments

In total, 124 independent SNPs were included as IVs for LSB. Among them, 98 SNPs were related to TV watching, 22 SNPs were for computer use, and four were for driving (Supplementary Table S1). In GWASs, the heritability was measured by the proportion of phenotypic variance explained by all SNPs (Zhu and Zhou, 2020). The heritability explained for leisure TV watching, leisure computer use, and leisure driving was 16.1%, 9.3%, and 4.4%, respectively, as estimated by van de Vegte *et al*. using the BOLT-REML analysis, which was available at https://www.hsph.harvard.edu/alkes-price/software/ (van de Vegte et al., 2020) (Table 3). Using the F-statistic, the combined strength of the instruments of the corresponding LSB was assessed, which ranged from 18,287.46 to 76,260.81 (Table 3), all greatly surpassing the limited value (*F* > 10) and further indicating the strong power of our selected genetic variants. Power calculations for the univariable IVW MR analyses performed using mRnd power calculator (Brion et al., 2013) indicated a 100% statistical power of TV watching. However, due to the low heritability of sedentary computer use and driving, the statistical power of computer use and driving remained scarce (< 80%) (Table 3). As estimated by van de Vegte *et al*., using the MR-Egger regression analysis, the IV of TV watching (*I* ^2^_GX_ = 0.98) and computer use (*I* ^2^_GX_ = 0.98) suggested a rare chance of weak-instrument bias, whereas driving (*I* ^2^_GX_ = 0) implied a high chance of weak-instrument bias. Consequently, the phenotypic between-group differences caused by genetic variation of driving were small, and the original confounded positive correlation was more likely to recover. The weak-instrument bias in the driving subgroup might influence the reliability of the results, and future GWASs with a larger sample size, which may explain more heritability of this phenotype, are warranted. The details of each SNP with potential pleiotropy for other traits are available in Supplementary Table S2.

**TABLE 3:** Power calculation for Mendelian randomization analysis.

Exposure/genetic instruments	R-square (of variance in phenotype)	Number of subjects	Number of cases	Proportion of cases	Observational OR	*F*-statistics	Power
Television watching/152 SNPs	0.161	400,364	12,136	0.030	1.20	76,260.81	1.00
Computer use/37 SNPs	0.093	397,641	12,085	0.030	0.97	40,773.45	0.17
Driving/four SNPs	0.044	397,315	12,081	0.030	1.03	18,287.46	0.10

### UVMR Analysis

**FIGURE 2:**
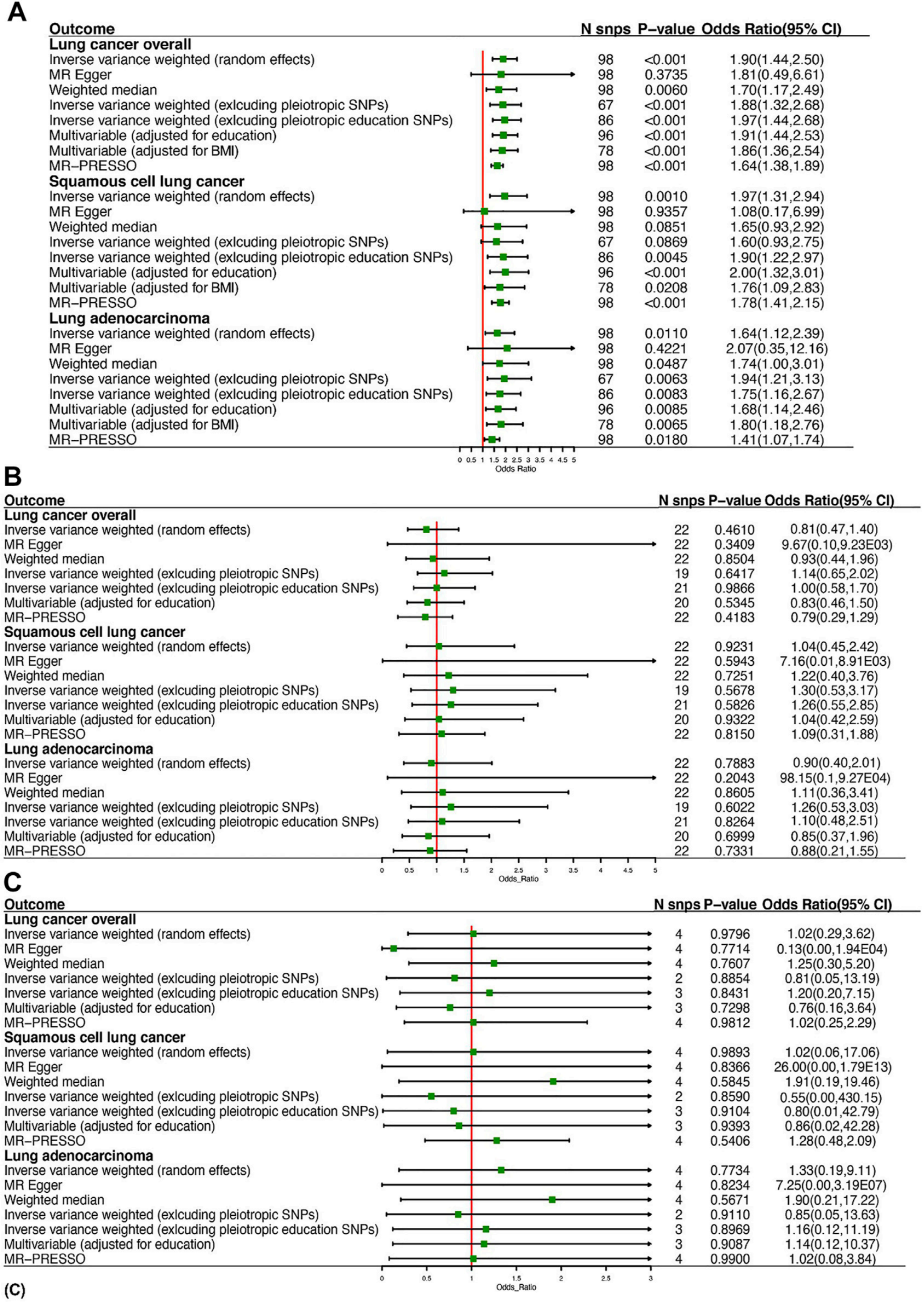
Summary of Mendelian randomization estimates of leisure sedentary behaviors on the risk of lung cancer: **(A)** is for watching television, **(B)** is for computer use, and **(C)** is for driving.

**FIGURE 3:**
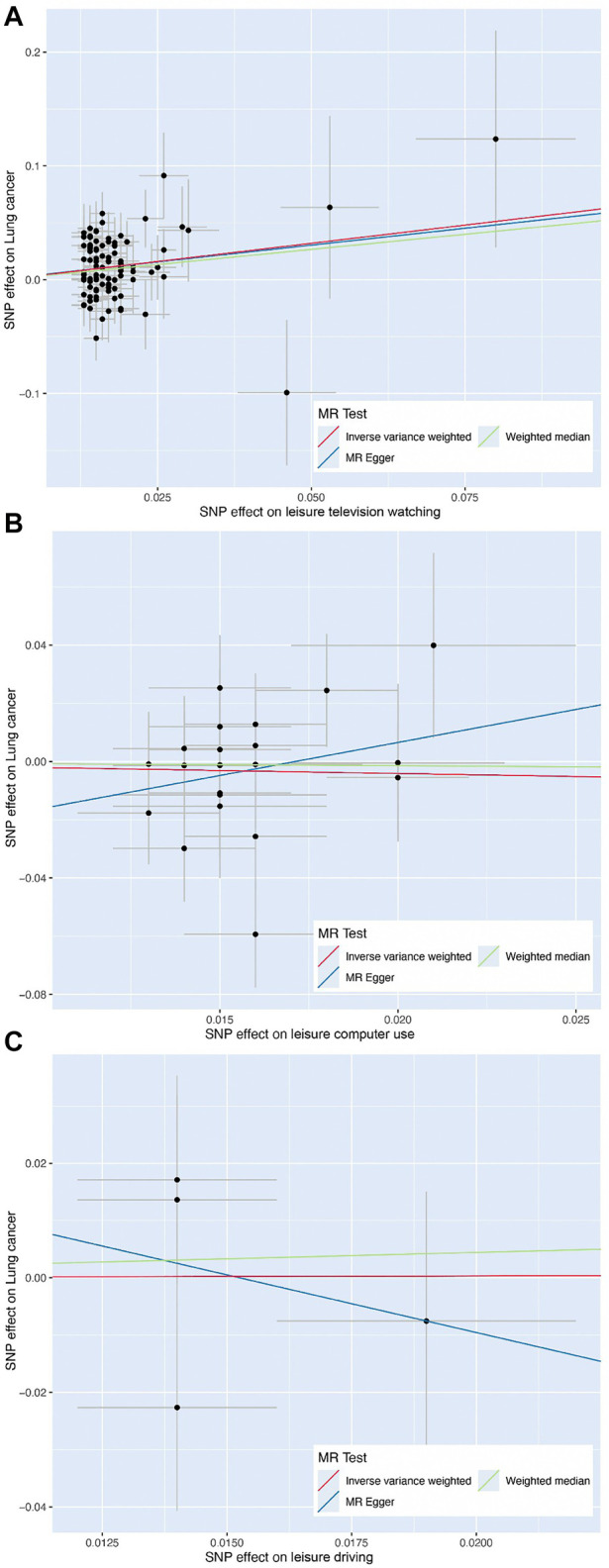
Scatter plot of the effect size of each single-nucleotide polymorphism on leisure television watching **(A)**, leisure computer use **(B)**, and leisure driving **(C)** and lung cancer risk using Mendelian randomization approaches, including inverse variance-weighted, weighted median, and MR-Egger, respectively.

Using 98 SNPs robustly associated with leisure TV watching, UVMR provided strong evidence that leisure TV watching increased the risk of LC overall (OR = 1.90, 95% CI = 1.44–2.50, *p* < 0.001) (Figures 2, 3). Given that our chosen SNPs for LSB were highly correlated with education, we excluded 12 pleiotropic education SNPs, and the same effect was observed in comparison to the gross IVW effect (OR = 1.97, 95% CI = 1.44–2.68, *p* < 0.001) (Figure 2). Additionally, after excluding pleiotropic SNPs, the IVW results from the remaining 67 SNPs appeared to be stable (OR = 1.88, 95% CI = 1.32–2.68, *p* < 0.001). The subgroup analyses demonstrated the same risky effects among both NSCLC subtypes, including LUSC (OR = 1.97, 95% CI = 1.31–2.94, *p* = 0.001) and LUAD (OR = 1.64, 95% CI = 1.12–2.39, *p* = 0.0110). The MR-Egger analysis did not demonstrate evidence of pleiotropy. Besides this, after excluding pleiotropic SNPs, the association between leisure TV watching and LUSC violated the result of the gross IVW method, which represented a null significant causation (OR = 1.60, 95% CI = 0.93–2.75, *p* = 0.0869), illustrating that the causal relationship between both phenotypes was not entirely determined by leisure TV watching itself (Figure 2) (Supplementary Table S3).

Findings of a causal effect of leisure computer use on overall LC risk using 22 SNPs suggested a null casual effect. In detail, per one SD increase in leisure time using computer was found to decrease the risk of LC, while the result remained not statistically significant (OR = 0.81, 95% CI = 0.47–1.40, *p* = 0.4610) (Figure 2). However, a potential increased risk of LC was shown after excluding pleiotropic SNPs (OR = 1.14, 95% CI = 0.65–2.02, *p* = 0.6417). In the subgroup analyses, the causal association between leisure computer use and LUAD remained the same as LC overall (OR = 0.90, 95% CI = 0.40–2.01, *p* = 0.7883). Although the result was insignificant, a slightly increased risk of LUSC was found instead (OR = 1.04, 95% CI = 0.45–2.42, *p* = 0.9231). Eventually, utilizing four SNPs independently associated with leisure driving, UVMR provided weak evidence that one-SD increase in leisure time driving elevated the risk of LC (OR = 1.02, 95% CI = 0.29–3.62, *p* = 0.9796) (Figure 2). The direction of effect was consistent across the subgroup analyses (OR = 1.02, 95% CI = 0.06–17.06, *p* = 0.9893 for LUSC, OR = 1.33, 95% CI = 0.19–9.11, *p* = 0.7734 for LUAD) (Figure 2). The Wald ratio estimate results of individual SNPs are described in Supplementary Table S4.

### MVMR Analysis

In the MVMR analysis controlling for education, there was strong evidence for a direct causal effect of per-SD increase in leisure time TV watching on the risk of LC (OR = 1.91, 95% CI = 1.44–2.53, *p* < 0.001) (Figure 2). In MVMR analysis controlling for BMI, strong evidence for a direct causal effect was observed between both phenotypes (OR = 1.86, 95% CI = 1.36–2.54, *p* < 0.001) (Figure 2). In MVMR analysis stratified by LC subtype, we found evidence for a causal independent effect of leisure TV watching in LUSC and LUAD after adjusting for education (OR = 2.00, 95% CI = 1.32–3.01, *p* < 0.001 for LUSC, OR = 1.68, 95% CI = 1.14–2.46, *p* = 0.0085 for LUAD) and BMI (OR = 1.76, 95% CI = 1.09–2.83, *p* = 0.0208 for LUSC, OR = 1.80, 95% CI = 1.18–2.76, *p* = 0.0065 for LUAD) (Figure 2).

We also investigated the direct effects between leisure computer use and LC using MVMR. Similar to the UVMR analyses, weak evidence for direct effects of leisure computer use adjusted for education on LC was observed as well as LUSC and LUAD. There was also no evidence for direct effects of leisure driving on LC, including LUAD and LUSC (Supplementary Table S3).

### Sensitivity Analysis

Regarding the correlations between LSB and risk of LC and subtypes, the directions of effect were mainly consistent in the weighted median method and MR-PRESSO test. At the same time, MR-Egger had wider CI and presented an insignificant effect, indicating a possible violation of the no measurement error assumption (Figure 2) (Zhou et al., 2021). Leave-one-out results indicated that no individual genetic variants appeared to significantly affect the overall results (Supplementary Figures 1–9).

As mentioned above, despite the casual relationship between leisure TV watching and risk of LC remaining robust after excluding pleiotropic SNPs, evidence of heterogeneity existed through Cochran’s Q test (Table 4; Supplementary Table S5), while MR-Egger intercepts indicated no evidence for directional pleiotropy (Table 4; Supplementary Table S6). No SNP effect outliers were detected from MR-PRESSO (Figure 2). Besides this, the abovementioned causation between computer use and LC remained conflicting before and after pleiotropic SNPs exclusion, potentially suggesting the existence of bias caused by the pleiotropy effects, while no evidence was supported among the heterogeneity test (Table 4; Supplementary Table S5), MR-Egger regression (Table 4; Supplementary Table S6), or MR-PRESSO outliner test (Supplementary Figure S2). In regard to *I* ^2^ statistic, we found that the heterogeneity across all leisure driving subgroups was significant while not observed in other subgroup analyses (Table 4).

**TABLE 4:** MR-Egger regression and heterogeneity analysis of the correlations between television watching, computer use, driving, and lung cancer risk.

Exposure	Heterogeneity *p*		MR-Egger regression		*I* ^2^ index
	MR-Egger	Inverse variance-weighted	Intercept	Intercept *p*	
TV watching	0.0391	0.0453	0.0008	0.9405	20.34%
TV watching (excluding pleiotropic single-nucleotide polymorphisms, SNPs)	0.0528	0.0610	0.0048	0.7217	22.01%
TV watching (excluding pleiotropic education SNPs)	0.0337	0.0394	0.0020	0.8708	22.19%
TV watching (adjusted for education)	0.0295	0.0344	0.0011	0.9238	21.00%
TV watching (adjusted for body mass index)	0.0492	0.0576	0.0010	0.9332	21.85%
Computer use	0.3659	0.3559	−0.0388	0.2968	7.81%
Computer use (excluding pleiotropic SNPs)	0.9439	0.9168	−0.0437	0.2298	0.00%
Computer use (excluding pleiotropic education SNPs)	0.9095	0.8804	−0.0431	0.2322	0.00%
Computer use (adjusted for education)	0.2623	0.2549	−0.0403	0.3154	15.97%
Driving	0.2274	0.3720	0.0307	0.7679	4.17%
Driving (excluding pleiotropic SNPs)	NA	0.1200	NA	NA	58.64%
Driving (excluding pleiotropic education SNPs)	NA	0.2274	NA	NA	32.47%
Driving (adjusted for education)	0.1200	0.2961	0.0102	0.9476	17.84%

### Mediating Effects

**FIGURE 4:**
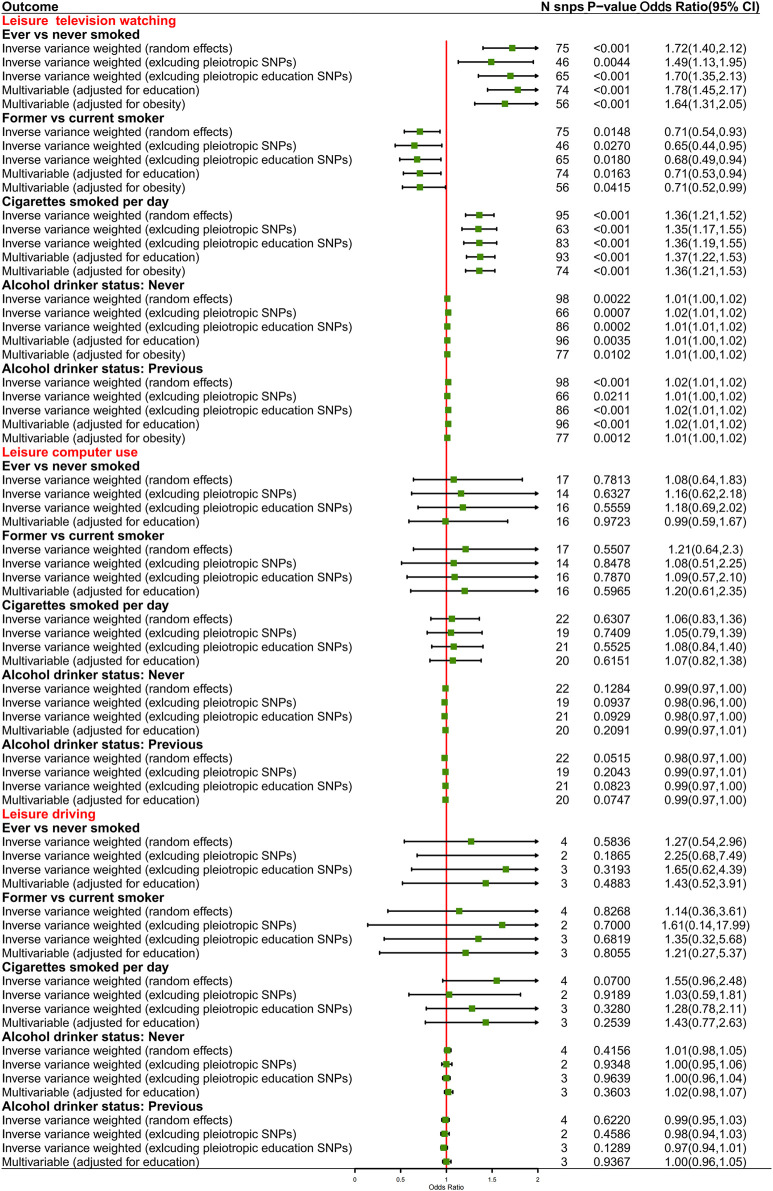
Causal effects from genetically predisposed prolonged leisure sedentary behaviors and mediators.

For exposure-mediator effects, we observed that genetically predisposed prolonged leisure TV watching was associated with smoking (OR 1.72, 95%CI 1.40–2.12, *p* < 0.001 for ever smokers; OR 1.36, 95%CI 1.21–1.52, *p* < 0.001 for cigarettes smoked per day; OR 0.71, 95%CI 0.54–0.93, *p* = 0.0148 for former smokers) and alcohol consumption (OR 1.02, 95%CI 1.01–1.02, *p* < 0.001 for previous drinkers; OR 1.01, 95%CI 1.00–1.02, *p* = 0.0022 for never drinkers), which was directionally consistent in the MVMR analyses. No significant causal associations between prolonged leisure computer use and driving and smoking and alcohol consumption were implied (Figure 4). For mediator-outcome effects, genetically predisposed smoking was positively associated with LC (OR 3.82, 95%CI 1.69–8.61, *p* = 0.0013 for ever smokers; OR 2.63, 95%CI 1.88–3.68, *p* < 0.001 for cigarettes smoked per day) and subtypes. Combining the above-mentioned findings, the causal effects from leisure TV watching to LC were partly mediated by smoking (Table 5).

**TABLE 5:** Causal effects from mediators to lung cancer and subtypes.

Exposures	Lung cancer		Squamous cell lung cancer		Lung adenocarcinoma	
	Causal effect (95% CI)	*p*-value	Causal effect (95% CI)	*p*-value	Causal effect (95% CI)	*p*-value
Ever *vs*. never smoked	3.82 (1.69, 8.61)	0.0013	4.93 (1.55, 15.68)	0.0068	5.46 (1.36, 21.96)	0.0168
Cigarettes smoked per day	2.63 (1.88, 3.68)	<0.001	3.10 (2.08, 4.63)	<0.001	2.49 (1.82, 3.41)	<0.001

## Discussion

In the present study, we utilized both UVMR and MVMR approaches to estimate a causal and independent effect of LSB on LC risk. We observed that genetically predisposed 1.5-h increase in LSB spent on watching TV was causally associated with increased risks of LC overall and among specific subtypes, including LUSC and LUAD, highlighting an important potential detrimental factor for public health. The causal effects remained to be significant after controlling for educational attainment and BMI. Similar magnitudes were not observed for prolonged LSB spent on computer use and driving. Genetically predisposed prolonged LSB was additionally associated with alcohol consumption and smoking. Moreover, our results were largely robust to several sensitivity analyses.

Our findings are in alignment with most of the previous observational studies. Indeed, the association between SB and risk of LC has been investigated across five large-scale cohort studies during the last decade. S Ukawa *et al.* found that the hazard ratio of LC for people who watched TV for more than 4 h per day increased by 36% compared with 2 h daily through the Japan Collaborative Cohort Study consisting of 54,258 adults, whereas the effect was only restricted to male rather than female individuals (Ukawa et al., 2013). Analogously, in 2016, a large-scale prospective cohort study including 129,401 postmenopausal women from the Women’s Health Initiative Observational Study did not support the positive correlation between SB and LC after adjusting for BMI, age, smoking, education, and so on (Wang et al., 2016). Moreover, this study also investigated whether SB would increase the odds of LC mortality, resulting in a null relationship. In contrast, a retrospective cohort study from Nord-Trøndelag Health Study (HUNT) reported that prolonged SB (≥8 h/day *vs*. <8 h/day) and low PA (≤8.3 MET-h/week *vs*. >16.6 MET-h/week) together were positively correlated with LC risk after controlling for age, education, smoking, and alcohol consumption, implying that the effect of SB on LC risk was unlikely to be independent of PA (Rangul et al., 2018). Later findings from HUNT further supported that prolonged SB was not independently correlated with the incidence of LC, whereas the combination of prolonged SB and physical inactivity increased the risk of LC overall, including NSCLC and small cell lung cancer (Jiang et al., 2019). Concerning occupational SB (OSB), the latest findings from the Japan Public Health Center-Based Prospective Study showed that it (≥7 h/day *vs*. 1 to <3 h/day) was connected with higher odds of LC by 180%; notably, this result only restricted to females, and it was nonsignificant for males (Ihira et al., 2020).

The limitations of previous observations cannot be ignored. First, a key shortcoming of all previous studies is that the measurement of SB was relied on questionnaires and self-reports rather than using objective quantitative monitors like accelerometers, which are extremely likely to be affected by the limitations of recall bias and high random error. Second, considering that SB traits are complex and a number of factors can impact SB, confounding bias cannot be fully avoided despite the fact that previous studies have made efforts in adjusting for confounders like BMI, smoking, and so on. Third, the sample size of LC patients was relatively limited in previous observational studies, with the largest consisting of 3,807 cases. Hence, it might be insufficient to provide adequate statistical power to evaluate the causal nexus. Furthermore, previous findings were likely to be affected by the intertwined relationship between PA and SB—for instance, people who have prolong SB tend to be physical inactive, whereas it does not necessarily mean so. Consequently, they could not fully disentangle the independent effects of SB on the risk of LC. Despite these shortcomings, the researchers have done a great job in probing the causal inference between SB and LC risk, with the largest cohort consisting of 54,258 participants (Ukawa et al., 2013) and the longest follow-up time being up to 18.3 years (Jiang et al., 2019).

The mechanisms underlying the attribution of SB to LC are under active investigation and, in the meantime, incompletely defined. Some animal studies demonstrated that SB might inhibit the activity of lipoprotein lipase in skeletal muscles and reduce glucose uptake, resulting in glucose dysregulation (Bey and Hamilton, 2003; Hamilton et al., 2004). Reduction in glucose consumption may subsequently cause hyperinsulinemia due to the consistently high levels of blood glucose. Meanwhile, increased concentrations of insulin-like growth factor-1, considered as the key regulator of energy metabolism and growth, were demonstrated to be modestly correlated with an increased risk of LC (Ho et al., 2016). Such metabolic disorder is connected with various cancer development (Wilmot et al., 2012). Moreover, several pre-clinical studies showed that weight-bearing skeletal muscles are not highly engaged during inactivity, which might suppress the anti-cancer responses of myokines themselves. The downregulation of anti-cancer responses may be a key activator for the inflammatory pathways characterized by the elevated concentrations of CRP, which are important for the development of several malignancies (Hojman et al., 2011; Pedersen and Febbraio, 2012; Aoi et al., 2013)**.** Moreover, scientists reported that SB may disrupt the balance between reactive oxygen species (ROS) and antioxidant defenses, resulting in the accumulation of ROS. ROS is known to cause DNA damage, chromosomal aberrations, and mutations, which inhibit the expression of tumor suppressor genes and increase the expression of oncogenes, attributing to cellular structure damage and further cancer development (Basu and Nohmi, 2018). Besides this, SB is often associated with snacking behaviors and obesity, while obesity has been positively correlated with a higher LC risk (Zhou et al., 2021). One emerging perspective is that obesity-related inflammation originating from the intestinal cavity could facilitate microbiome-producing cancer-promoting metabolites and afterward leak them into the bloodstream for the target organ (Mao et al., 2018; Zhuang et al., 2019). Our study also provided evidence that the prolonged time spent on LSB was positively correlated with smoking and alcohol consumption, further elucidating the potential mediating mechanisms from LSB to LC.

The notable strengths of our study are as follows: First, with the large-scale summary genetic data from UK Biobank (up to 422,218 individuals) and robustly correlated SNPs as IV (F statistics >10), our study had adequate statistical power for probing the causal effect between LSB and risk of LC for the first time; second, using the MVMR approach adjusted for educational attainment and BMI, we were more likely to describe a relatively independent causal inference from LSB to LC. Thirdly, a number of pleiotropy-robust MR analyses and outlier detection (weighted median, MR-Egger, and MR-PRESSO) were performed to rigorously investigate whether the IVW estimates were biased due to pleiotropy, resulting in mostly unbiased results. Besides this, using MR design, our findings are less likely to be affected by measurement error than conventional observational studies, of which the data for SB were subjectively measured and hence underestimated (Urda et al., 2017). Finally, to the best of our knowledge, we first explored the associations between LSB and different histology subtypes of LC, which may be more generalizable under different conditions.

Understanding the limitations of our study means to interpret the findings better. First, given that OSB accounts for an important part of SB in our daily life, our findings cannot be generalized to total SB for the reason that the GWAS we used did not include genetic data for OSB. Secondly, the ascertainment of SB time in the GWAS was evaluated through questionnaires, and hence recall bias cannot be fully excluded in our study either. In addition, the SNPs we used and the ILCCO consortia included were made of European descent. Consequently, whether our conclusions can be generalized to other ethnicities and regions remains unclear. Additionally, we observed evidence for heterogeneity, and hence potential pleiotropy may occur in the MVMR analysis even controlling for education and BMI, indicating that unobserved confounders may affect the association between watching TV and LC. Therefore, we advocate that researchers interpret our findings with caution, and further analyses account for pleiotropy should be conducted when the methods are developed. Moreover, despite that we have calculated the F-statistic of UVMR analysis, it is currently impossible to estimate the F-statistic for MVMR considering that the SNP effect on the other exposure is taken into account. Therefore, we were unable to analyze the strength of IV for each exposure. Finally, we were incapable of performing subgroup analyses stratified by age, socioeconomic status, and other covariates of interest, which rely on individual-level data that we did not have access to.

Considering that all of our MR results were in the same direction, it seems to support that prolonged SB spent on watching TV is a potential risk factor for LC. Sedentary TV watching ranks as the most prevalent SB, and almost 90% of older American adults (age >60 years) watch TV with an average of 4.7 h per day (Matthews et al., 2008). Regarding young adults (ages 20–29 years), they tend to spend prolonged time watching TV at night for an average of 3.6 h, usually followed by OSB at daytime (Neuhaus et al., 2014). Given the previously identified incidence risks, all-cause mortality risks of LC, and high prevalence and the discretionary nature of watching TV, reducing such behavior may substantially impact public health. LM León-Muñoz *et al*. have reported that people who remain non-sedentary (median sedentary time <3 h/day) had 25% lower mortality than the consistently sedentary ones (median sedentary time >7 h/day) on the basis of a prospective cohort including 2,635 persons (León-muñoz et al., 2013), supported by J Lee *et al*. that post-menopausal women who spent fewer time on sitting were correlated with 48% lower risk of cancer mortality (Lee et al., 2016). In addition to the lower mortality, reducing TV watching time may potentially increase the overall PA. A randomized controlled trial found that a 2.- h/day reduction in watching TV contributed to an increase in the objectively measured PA of 119 kcal/day (Otten et al., 2009). Meanwhile, consisting of 20 cohort studies and six case–control studies, the latest meta-analysis also demonstrated that regular leisure time PA was associated with 24% reduced risk of LC (Brenner et al., 2016). The Physical Activity Guidelines for Americans also recommend for people to replace SB with light-intensity or moderate-intensity PA, which are beneficial for almost all ages (Piercy et al., 2018; Ekelund et al., 2020). Interestingly, the latter MR study did not support the causality between PA and LC (Xian et al., 2021). In consideration of the tangled relationship among SB, PA, and LC, further studies should pay more attention to defining the specific type and intensity of PA and SB to have a deeper understanding of the mechanisms underlying these associations.

## Conclusion

Collectively, our study provided evidence for a causal relationship between SB as measured by watching TV and the increased risks of LC overall and among specific subtypes for LUAD and LUSC. Our findings may provide oncologists and public health sanitarians with new evidence to support interventions on behavior to reduce sedentary TV viewing, possibly by lowering the incidence and mortality of LC at a population level. Further studies with robust measurements, large-scale sample size, sufficient genetic data, and more effective MR approaches are needed to better understand both the behavioral and biological mechanisms that mediate the associations between domain-specific SB and risks of LC.

## Data Availability

The original contributions presented in the study are included in the article/supplementary material. Further inquiries can be directed to the corresponding authors.

## Abbreviations

BMI, body mass index; CI, confidence interval; CRP, c-reactive protein; GWASs, genome-wide association studies; HR, hazard ratio; ILCCO, international lung cancer consortium; IV, instrumental variable; IVW, inverse variance-weighted; LC, lung cancer; LD, linkage disequilibrium; LSB, leisure sedentary behavior; LUAD, lung adenocarcinoma; LUSC, squamous cell lung cancer; MR, mendelian randomization; MR-PRESSO, MR pleiotropy residual sum and outlier; METs, metabolic equivalents; MVMR, multivariable mendelian randomization; NSCLC, non-small cell lung cancer; NOME, no measurement error; OR, odds ratio; OSB, occupational sedentary behavior; PA, physical activity; ROS, reactive oxygen species; RCT, randomized controlled trial; SB, sedentary behavior; SD, standard deviation; SNP, single-nucleotide polymorphism; TV, television; UVMR, univariable mendelian randomization.

## References

[B1] Aoi W. Naito Y. Takagi T. Tanimura Y. Takanami Y. Kawai Y. (2013). A Novel Myokine, Secreted Protein Acidic and Rich in Cysteine (SPARC), Suppresses colon Tumorigenesis via Regular Exercise. Gut 62 (6), 882–889. 10.1136/gutjnl-2011-300776 22851666

[B2] Basterra-Gortari F. J. Bes-Rastrollo M. Gea A. Núñez-Córdoba J. M. Toledo E. Martínez-González M. Á. (2014). Television Viewing, Computer Use, Time Driving and All-Cause Mortality: the SUN Cohort. J. Am. Heart Assoc. 3 (3), e000864. 10.1161/JAHA.114.000864 24965030 PMC4309083

[B3] Basu A. K. Nohmi T. (2018). Chemically-Induced DNA Damage, Mutagenesis, and Cancer. Int. J. Mol. Sci. 19 (4), 970. 10.3390/ijms19040970 PMC6032311 29899224

[B4] Bey L. Hamilton M. T. (2003). Suppression of Skeletal Muscle Lipoprotein Lipase Activity during Physical Inactivity: a Molecular Reason to Maintain Daily Low-Intensity Activity. J. Physiol. 551 (Pt 2), 673–682. 10.1113/jphysiol.2003.045591 12815182 PMC2343229

[B5] Bowden J. Davey Smith G. Burgess S. (2015). Mendelian Randomization with Invalid Instruments: Effect Estimation and Bias Detection through Egger Regression. Int. J. Epidemiol. 44 (2), 512–525. 10.1093/ije/dyv080 26050253 PMC4469799

[B6] Bowden J. Del Greco M F. Minelli C. Davey Smith G. Sheehan N. Thompson J. (2017). A Framework for the Investigation of Pleiotropy in Two-Sample Summary Data Mendelian Randomization. Statist. Med. 36 (11), 1783–1802. 10.1002/sim.7221 PMC5434863 28114746

[B7] Brenner D. R. Yannitsos D. H. Farris M. S. Johansson M. Friedenreich C. M. (2016). Leisure-time Physical Activity and Lung Cancer Risk: A Systematic Review and Meta-Analysis. Lung Cancer 95, 17–27. 10.1016/j.lungcan.2016.01.021 27040847

[B8] Brion M.-J. A. Shakhbazov K. Visscher P. M. (2013). Calculating Statistical Power in Mendelian Randomization Studies. Int. J. Epidemiol. 42 (5), 1497–1501. 10.1093/ije/dyt179 24159078 PMC3807619

[B9] Burgess S. Thompson S. G. (2015). Multivariable Mendelian Randomization: the Use of Pleiotropic Genetic Variants to Estimate Causal Effects. Am. J. Epidemiol. 181 (4), 251–260. 10.1093/aje/kwu283 25632051 PMC4325677

[B10] Carter B. D. Abnet C. C. Feskanich D. Freedman N. D. Hartge P. Lewis C. E. (2015). Smoking and Mortality - beyond Established Causes. N. Engl. J. Med. 372 (7), 631–640. 10.1056/nejmsa1407211 25671255

[B11] Censin J. C. Peters S. A. E. Bovijn J. Ferreira T. Pulit S. L. Mägi R. (2019). Causal Relationships between Obesity and the Leading Causes of Death in Women and Men. Plos Genet. 15 (10), e1008405. 10.1371/journal.pgen.1008405 31647808 PMC6812754

[B12] Ekelund U. Tarp J. Fagerland M. W. Johannessen J. S. Hansen B. H. Jefferis B. J. (2020). Joint Associations of Accelerometer-Measured Physical Activity and Sedentary Time with All-Cause Mortality: a Harmonised Meta-Analysis in More Than 44 000 Middle-Aged and Older Individuals. Br. J. Sports Med. 54 (24), 1499–1506. 10.1136/bjsports-2020-103270 33239356 PMC7719907

[B13] Emdin C. A. Khera A. V. Kathiresan S. (2017). Mendelian Randomization. JAMA 318 (19), 1925–1926. 10.1001/jama.2017.17219 29164242

[B14] Greco M F. D. Minelli C. Sheehan N. A. Thompson J. R. (2015). Detecting Pleiotropy in Mendelian Randomisation Studies with Summary Data and a Continuous Outcome. Statist. Med. 34 (21), 2926–2940. 10.1002/sim.6522 25950993

[B15] Hamilton M. T. Hamilton D. G. Zderic T. W. (2004). Exercise Physiology versus Inactivity Physiology: an Essential Concept for Understanding Lipoprotein Lipase Regulation. Exerc. Sport Sci. Rev. 32 (4), 161–166. 10.1097/00003677-200410000-00007 15604935 PMC4312662

[B16] Hemani G. Zheng J. Elsworth B. Wade K. H. Haberland V. Baird D. (2018). The MR-Base Platform Supports Systematic Causal Inference across the Human Phenome. Elife 7. 10.7554/eLife.34408 PMC5976434 29846171

[B17] Ho G. Y. F. Zheng S. L. Cushman M. Perez-Soler R. Kim M. Xue X. (2016). Associations of Insulin and IGFBP-3 with Lung Cancer Susceptibility in Current Smokers. J. Natl. Cancer Inst. 108 (7). 10.1093/jnci/djw012 PMC5929107 27071409

[B18] Hojman P. Dethlefsen C. Brandt C. Hansen J. Pedersen L. Pedersen B. K. (2011). Exercise-induced Muscle-Derived Cytokines Inhibit Mammary Cancer Cell Growth. Am. J. Physiology-Endocrinology Metab. 301 (3), E504–E510. 10.1152/ajpendo.00520.2010 21653222

[B19] Ihira H. Sawada N. Yamaji T. Goto A. Shimazu T. Kikuchi H. (2020). Occupational Sitting Time and Subsequent Risk of Cancer: The Japan Public Health Center‐based Prospective Study. Cancer Sci. 111 (3), 974–984. 10.1111/cas.14304 31925977 PMC7060463

[B20] Jiang L. Sun Y.-Q. Brumpton B. M. Langhammer A. Chen Y. Nilsen T. I. L. (2019). Prolonged Sitting, its Combination with Physical Inactivity and Incidence of Lung Cancer: Prospective Data from the HUNT Study. Front. Oncol. 9, 101. 10.3389/fonc.2019.00101 30859092 PMC6397867

[B21] Katzmarzyk P. T. Powell K. E. Jakicic J. M. Troiano R. P. Piercy K. Tennant B. (2019). Sedentary Behavior and Health: Update from the 2018 Physical Activity Guidelines Advisory Committee. Med. Sci. Sports Exerc. 51 (6), 1227–1241. 10.1249/mss.0000000000001935 31095080 PMC6527341

[B22] Kerr J. Anderson C. Lippman S. M. (2017). Physical Activity, Sedentary Behaviour, Diet, and Cancer: an Update and Emerging New Evidence. Lancet Oncol. 18 (8), e457–e471. 10.1016/s1470-2045(17)30411-4 28759385 PMC10441558

[B23] Larsson S. C. Carter P. Kar S. Vithayathil M. Mason A. M. Michaëlsson K. (2020). Smoking, Alcohol Consumption, and Cancer: A Mendelian Randomisation Study in UK Biobank and International Genetic Consortia Participants. Plos Med. 17 (7), e1003178. 10.1371/journal.pmed.1003178 32701947 PMC7377370

[B24] Lawlor D. A. Harbord R. M. Sterne J. A. C. Timpson N. Davey Smith G. (2008). Mendelian Randomization: Using Genes as Instruments for Making Causal Inferences in Epidemiology. Statist. Med. 27 (8), 1133–1163. 10.1002/sim.3034 17886233

[B25] Lee J. Kuk J. L. Ardern C. I. (2016). The Relationship between Changes in Sitting Time and Mortality in post-menopausal US Women. J. Public Health 38 (2), 270–278. 10.1093/pubmed/fdv055 PMC4894483 25935896

[B26] León-muñoz L. M. Martínez-gómez D. Balboa-Castillo T. López-garcía E. Guallar-castillón P. Rodríguez-artalejo F. (2013). Continued Sedentariness, Change in Sitting Time, and Mortality in Older Adults. Med. Sci. Sports Exerc. 45 (8), 1501–1507. 10.1249/mss.0b013e3182897e87 23439420

[B27] Mao Q. Jiang F. Yin R. Wang J. Xia W. Dong G. (2018). Interplay between the Lung Microbiome and Lung Cancer. Cancer Lett. 415, 40–48. 10.1016/j.canlet.2017.11.036 29197615

[B28] Matthews C. E. Chen K. Y. Freedson P. S. Buchowski M. S. Beech B. M. Pate R. R. (2008). Amount of Time Spent in Sedentary Behaviors in the United States, 2003-2004. Am. J. Epidemiol. 167 (7), 875–881. 10.1093/aje/kwm390 18303006 PMC3527832

[B29] Neuhaus M. Eakin E. G. Straker L. Owen N. Dunstan D. W. Reid N. (2014). Reducing Occupational Sedentary Time: a Systematic Review and Meta-Analysis of Evidence on Activity-Permissive Workstations. Obes. Rev. 15 (10), 822–838. 10.1111/obr.12201 25040784

[B30] Otten J. J. Jones K. E. Littenberg B. Harvey-Berino J. (2009). Effects of Television Viewing Reduction on Energy Intake and Expenditure in Overweight and Obese Adults. Arch. Intern. Med. 169 (22), 2109–2115. 10.1001/archinternmed.2009.430 20008695

[B31] Pedersen B. K. Febbraio M. A. (2012). Muscles, Exercise and Obesity: Skeletal Muscle as a Secretory Organ. Nat. Rev. Endocrinol. 8 (8), 457–465. 10.1038/nrendo.2012.49 22473333

[B32] Phelan C. M. Kuchenbaecker K. B. Tyrer J. P. Kar S. P. Lawrenson K. Winham S. J. (2017). Identification of 12 New Susceptibility Loci for Different Histotypes of Epithelial Ovarian Cancer. Nat. Genet. 49 (5), 680–691. 10.1038/ng.3826 28346442 PMC5612337

[B33] Piercy K. L. Troiano R. P. Ballard R. M. Carlson S. A. Fulton J. E. Galuska D. A. (2018). The Physical Activity Guidelines for Americans. JAMA 320 (19), 2020–2028. 10.1001/jama.2018.14854 30418471 PMC9582631

[B34] Rangul V. Sund E. R. Mork P. J. Røe O. D. Bauman A. (2018). The Associations of Sitting Time and Physical Activity on Total and Site-specific Cancer Incidence: Results from the HUNT Study, Norway. PLoS ONE 13 (10), e0206015. 10.1371/journal.pone.0206015 30352079 PMC6198967

[B35] Sanderson E. Spiller W. Bowden J. (2021). Testing and Correcting for Weak and Pleiotropic Instruments in Two-Sample Multivariable Mendelian Randomization. Stat. Med. 40 (25), 5434–5452. 10.1002/sim.9133 34338327 PMC9479726

[B36] Sanderson E. Davey Smith G. Windmeijer F. Bowden J. (2019). An Examination of Multivariable Mendelian Randomization in the Single-Sample and Two-Sample Summary Data Settings. Int. J. Epidemiol. 48 (3), 713–727. 10.1093/ije/dyy262 30535378 PMC6734942

[B37] Schmid D. Leitzmann M. F. (2014). Television Viewing and Time Spent Sedentary in Relation to Cancer Risk: a Meta-Analysis. J. Natl. Cancer Inst. 106 (7). 10.1093/jnci/dju098 24935969

[B38] Schmid D. Ricci C. Behrens G. Leitzmann M. F. (2016). Does Smoking Influence the Physical Activity and Lung Cancer Relation? A Systematic Review and Meta-Analysis. Eur. J. Epidemiol. 31 (12), 1173–1190. 10.1007/s10654-016-0186-y 27502335

[B39] Sekula P. Del Greco M F. Pattaro C. Köttgen A. (2016). Mendelian Randomization as an Approach to Assess Causality Using Observational Data. Jasn 27 (11), 3253–3265. 10.1681/asn.2016010098 27486138 PMC5084898

[B40] Siegel R. L. Miller K. D. Fuchs H. E. Jemal A. (2021). Cancer Statistics, 2021. CA Cancer J. Clin. 71 (1), 7–31. 10.3322/caac.21654 33433946

[B41] Smith G. D. Ebrahim S. (2004). Mendelian Randomization: Prospects, Potentials, and Limitations. Int. J. Epidemiol. 33 (1), 30–42. 10.1093/ije/dyh132 15075143

[B42] Swerdlow D. I. Kuchenbaecker K. B. Shah S. Sofat R. Holmes M. V. White J. (2016). Selecting Instruments for Mendelian Randomization in the Wake of Genome-wide Association Studies. Int. J. Epidemiol. 45 (5), 1600–1616. 10.1093/ije/dyw088 27342221 PMC5100611

[B43] Ukawa S. Tamakoshi A. Wakai K. Noda H. Ando M. Iso H. (2013). Prospective Cohort Study on Television Viewing Time and Incidence of Lung Cancer: Findings from the Japan Collaborative Cohort Study. Cancer Causes Control 24 (8), 1547–1553. 10.1007/s10552-013-0231-z 23686441

[B44] Urda J. L. Larouere B. Verba S. D. Lynn J. S. (2017). Comparison of Subjective and Objective Measures of Office Workers' Sedentary Time. Prev. Med. Rep. 8, 163–168. 10.1016/j.pmedr.2017.10.004 29062680 PMC5645177

[B45] van de Vegte Y. J. Said M. A. Rienstra M. van der Harst P. Verweij N. (2020). Genome-wide Association Studies and Mendelian Randomization Analyses for Leisure Sedentary Behaviours. Nat. Commun. 11 (1), 1770. 10.1038/s41467-020-15553-w 32317632 PMC7174427

[B46] Verbanck M. Chen C.-Y. Neale B. Do R. (2018). Publisher Correction: Detection of Widespread Horizontal Pleiotropy in Causal Relationships Inferred from Mendelian Randomization between Complex Traits and Diseases. Nat. Genet. 50 (8), 1196. 10.1038/s41588-018-0164-2 29967445

[B47] Wang A. Qin F. Hedlin H. Desai M. Chlebowski R. Gomez S. (2016). Physical Activity and Sedentary Behavior in Relation to Lung Cancer Incidence and Mortality in Older Women: The Women's Health Initiative. Int. J. Cancer 139 (10), 2178–2192. 10.1002/ijc.30281 27439221 PMC5501309

[B48] Wang Y. McKay J. D. Rafnar T. Wang Z. Timofeeva M. N. Broderick P. (2014). Rare Variants of Large Effect in BRCA2 and CHEK2 Affect Risk of Lung Cancer. Nat. Genet. 46 (7), 736–741. 10.1038/ng.3002 24880342 PMC4074058

[B49] Wilmot E. G. Edwardson C. L. Achana F. A. Davies M. J. Gorely T. Gray L. J. (2012). Sedentary Time in Adults and the Association with Diabetes, Cardiovascular Disease and Death: Systematic Review and Meta-Analysis. Diabetologia 55 (11), 2895–2905. 10.1007/s00125-012-2677-z 22890825

[B50] Xian W. Shen J. Zhou H. Liu J. Zhang Y. Zhang Z. (2021). Mendelian Randomization Study Indicates Lack of Causal Relationship between Physical Activity and Lung Cancer. J. Cancer Res. Clin. Oncol. 147 (1), 177–181. 10.1007/s00432-020-03409-1 32989605 PMC11801823

[B51] Yang J. J. Yu D. Xiang Y.-B. Blot W. White E. Robien K. (2020). Association of Dietary Fiber and Yogurt Consumption with Lung Cancer Risk. JAMA Oncol. 6 (2), e194107. 10.1001/jamaoncol.2019.4107 31647500 PMC6813596

[B52] Zhou W. Liu G. Hung R. J. Haycock P. C. Aldrich M. C. Andrew A. S. (2021). Causal Relationships between Body Mass index, Smoking and Lung Cancer: Univariable and Multivariable Mendelian Randomization. Int. J. Cancer 148 (5), 1077–1086. 10.1002/ijc.33292 32914876 PMC7845289

[B53] Zhu H. Zhou X. (2020). Statistical Methods for SNP Heritability Estimation and Partition: A Review. Comput. Struct. Biotechnol. J. 18, 1557–1568. 10.1016/j.csbj.2020.06.011 32637052 PMC7330487

[B54] Zhuang H. Cheng L. Wang Y. Zhang Y.-K. Zhao M.-F. Liang G.-D. (2019). Dysbiosis of the Gut Microbiome in Lung Cancer. Front. Cel. Infect. Microbiol. 9, 112. 10.3389/fcimb.2019.00112 PMC6489541 31065547

