# Supplementary material for: Univariable and Multivariable Two-Sample Mendelian Randomization Investigating the Effects of Leisure Sedentary Behaviors on the Risk of Lung Cancer
Source: Front Genet. 2021 Nov 24;12:742718. doi: 10.3389/fgene.2021.742718 (PMC8651878; doi:10.3389/fgene.2021.742718)
Supplement: Supplementary file 1 [file DataSheet1.DOCX]

**Supplementary figure legends**

Supplementary figure 1. Leave-one-out analysis for causal association between leisure television watching and risk of lung cancer.

Supplementary figure 2. Leave-one-out analysis for causal association between leisure television watching and risk of squamous cell lung cancer.

Supplementary figure 3. Leave-one-out analysis for causal association between leisure television watching and risk of lung adenocarcinoma.

Supplementary figure 4. Leave-one-out analysis for causal association between leisure computer use and risk of lung cancer.

Supplementary figure 5. Leave-one-out analysis for causal association between leisure computer use and risk of squamous cell lung cancer.

Supplementary figure 6. Leave-one-out analysis for causal association between leisure computer use and risk of lung adenocarcinoma.

Supplementary figure 7. Leave-one-out analysis for causal association between leisure driving and risk of lung cancer.

Supplementary figure 8. Leave-one-out analysis for causal association between leisure driving and risk of squamous cell lung cancer.

Supplementary figure 9. Leave-one-out analysis for causal association between leisure driving and risk of lung adenocarcinoma.

Supplementary figure 1. Leave-one-out analysis for causal association between leisure television watching and risk of lung cancer.

Supplementary figure 2. Leave-one-out analysis for causal association between leisure television watching and risk of squamous cell lung cancer.

Supplementary figure 3. Leave-one-out analysis for causal association between leisure television watching and risk of lung adenocarcinoma.

Supplementary figure 4. Leave-one-out analysis for causal association between leisure computer use and risk of lung cancer.

Supplementary figure 5. Leave-one-out analysis for causal association between leisure computer use and risk of squamous cell lung cancer.

Supplementary figure 6. Leave-one-out analysis for causal association between leisure computer use and risk of lung adenocarcinoma.

Supplementary figure 7. Leave-one-out analysis for causal association between leisure driving and risk of lung cancer.

Supplementary figure 8. Leave-one-out analysis for causal association between leisure driving and risk of squamous cell lung cancer.

Supplementary figure 9. Leave-one-out analysis for causal association between leisure driving and risk of lung adenocarcinoma.
